# Mental health in people living with and beyond colorectal cancer: A patient‐oriented constructivist grounded theory

**DOI:** 10.1002/cam4.70203

**Published:** 2024-09-05

**Authors:** Vicki Cheng, Helen McTaggart‐Cowan, Jonathan M. Loree, Rachel A. Murphy, Mikaela Barnes, Haydn Bechthold, Norman Jansen, Mary A. De Vera

**Affiliations:** ^1^ Faculty of Pharmaceutical Sciences University of British Columbia Vancouver British Columbia Canada; ^2^ Collaboration for Outcomes Research and Evaluation Vancouver British Columbia Canada; ^3^ Cancer Control Research, BC Cancer Vancouver British Columbia Canada; ^4^ Faculty of Health Sciences Simon Fraser University Burnaby British Columbia Canada; ^5^ Division of Medical Oncology, Department of Medicine, Faculty of Medicine University of British Columbia Vancouver British Columbia Canada; ^6^ School of Population and Public Health University of British Columbia Vancouver British Columbia Canada; ^7^ Patient Research Partner Vancouver British Columbia Canada; ^8^ Centre for Health Evaluation and Outcome Sciences University of British Columbia Vancouver British Columbia Canada

**Keywords:** colorectal cancer, experiences, mental health, patient‐centered, qualitative research

## Abstract

**Background:**

With the burden of colorectal cancer in Canada, there is a need to address the psycho‐oncologic challenges, including mental health. This study aims to explore the lived mental health experiences in patients with CRC across the phases of the CRC care continuum.

**Methods:**

We employed a patient‐oriented constructivist grounded theory design and recruited English speaking participants ≥18 years, diagnosed with CRC within the last 10 years, residing in Canada. We collected data through semi‐structured individual interviews using a guide co‐constructed with patient research partners. Data collection and analysis were iterative, employed theoretical sampling, and culminated in a theoretical model.

**Results:**

Twenty‐eight participants diagnosed with CRC (18 females, 10 males), aged 18–63 years at time of diagnosis were interviewed, with representation across all CRC stages. There were 10 participants (36%) in treatment, 12 participants (43%) in follow‐up, and 6 participants (21%) in the beyond phase. We constructed a patient‐oriented theory illustrating the dynamic nature between one's self‐identity and their mental health experiences across the CRC care continuum. Mental health experiences encompass emotional and cognitive‐behavioral responses, expressed differently across phases. Mental health care experiences are also shaped by barriers, facilitators, and individual contextual factors, all of which influence their access to care.

**Conclusion:**

Our theory provides insight into the mental health experiences of patients with CRC across phases of the CRC care continuum. Understanding patients' emotional and cognitive‐behavioral responses and care experiences can help identify opportunities to integrate mental health into CRC care.

## 1 INTRODUCTION

A diagnosis of colorectal cancer (CRC) is associated with substantial physical stressors, including surgery, chemotherapy, radiation, and many times, even the creation of an ostomy.^1^, ^2^ Patients with a CRC diagnosis also report increased limitations of physical functioning, such as the inability to do housework, walk a half mile, or walk up and down stairs, compared to the general population.^3^ The physical impacts of CRC are well established, and in recent years, there has been a growing interest in the mental health impacts of this diagnosis.

Mental health, “a state of mental well‐being,” encompasses an individual's emotional, behavioral, and social well‐being, affecting one's feelings, behaviors, thoughts and actions.^4^, ^5^, ^6^ As such, mental health exists on a complex continuum and is experienced uniquely by individuals through varying levels of emotional and cognitive symptoms.^7^, ^8^ Therefore, understanding experiences of patients with CRC with mental health necessitates a qualitative inquiry. A few qualitative studies have explored patients with CRC's experiences throughout different stages of cancer care, including pre‐operative,^9^ operative,^10^, ^11^ postoperative^12^ and survivorship.^13^, ^14^ A 2022 qualitative study exploring pre‐surgery experiences in CRC patients revealed that although rarely addressed, mental and emotional health were particularly important to participants in the pre‐operative stage.^9^ A 2018 grounded theory study found that all 24 participants undergoing treatment experienced distress (i.e., a negative emotional reaction) both preoperatively and operatively.^11^ Furthermore, a 2022 narrative review on the needs of patients and survivors with CRC found that they have diverse needs, including informational, psychological, social, physical, financial, spiritual, with psychological and informational needs being the most important among patients.^15^ However, existing literature have not explicitly focused on the overall mental health experiences or access to mental health care for patients with CRC throughout the entire care trajectory. As such, we aimed to develop a patient‐oriented constructivist grounded theory to understand and contextualize mental health experiences from the patient perspective at every phase of the CRC care continuum (treatment, follow‐up, beyond), including their access to formal (professional) and informal (family, community) types of support within the Canadian healthcare system.

## 2 METHODS

### 2.1 Design

Our study was guided by a patient‐oriented constructivist grounded theory approach. Patient‐oriented research involves actively engaging patients as research partners throughout the process, shifting from the traditional participant role and allowing patients with CRC to be valuable contributors to the research.^16^, ^17^ Our patient research partners (MB, HB, NJ) were involved throughout the research process, including interview guide development, recruitment, data interpretation, and knowledge translation. Constructivist grounded theory is a qualitative research methodology that aims to explain human phenomena by generating a theory and acknowledges the researcher and participants as co‐constructors of its meaning.^18^, ^19^, ^20^, ^21^, ^22^, ^23^ Constructivist grounded theory, developed by Charmaz,^22^ uses the comparative, emergent, inductive, and open‐ended approach, inviting the researcher as a co‐constructor of the meaning of the studied phenomenon.^22^, ^23^ Constructivist grounded theory adds reflexivity, acknowledging the researcher's role in conducting the research. We integrated this approach, which acknowledges the subjectivity of researchers and participants, with patient‐oriented research, to comprehensively describe the phenomenon of CRC and mental health and gain a better understanding of patients' mental health experiences throughout the care continuum. This study was approved by the Behavioral Research Ethics Board at the University of British Columbia.

### 2.2 Setting and participants

The setting of the study is Vancouver, British Columbia, which is situated within a publicly funded healthcare system. We recruited participants using physical posters and pamphlets at British Columbia Cancer sites in Vancouver and social media posts through investigator's and patient organizations' channels. Individuals with CRC were eligible if they were: (1) age 18 years or older; (2) diagnosed with CRC in the last 10 years; (3) reside in Canada; and (4) able to communicate in English. Participants, upon expressing interest, accessed the study URL to electronically complete the consent form and a brief demographics questionnaire that includes the Hospital Anxiety and Depression Scale (HADS) questionnaire^24^ on the online survey platform, Qualtrics.^25^ The HADS questionnaire is an efficacious screening instrument for evaluating levels of anxiety and depression. All information collected aided in scheduling, tailoring the interview guide to each participant's CRC experiences, and assessing their baseline mental health levels through HADS scores at different phases of the CRC care continuum. We purposively sampled participants^20^ to ensure diversity with respect to CRC stage, phase of care and HADS scores, with our sample size pragmatically influenced by participant willingness and availability.

### 2.3 Data collection

One‐to‐one semi‐structured interviews (~1 h) were conducted virtually over the video‐conferencing platform, Zoom. Interviews were digitally recorded and transcribed using a professional online transcription software, Sonix.^26^ Data collection and analysis occurred simultaneously, with ongoing iterative processes where analysis informed subsequent data collection, continuing until thematic saturation was achieved.^20^ In our study, we operationalized thematic analysis by adopting the concept of “theoretical sufficiency,” which focuses on achieving adequate depth of understanding to build a theory or generate relevant themes.^27^ Therefore, we prioritized data richness and diversity over quantity, ensuring that our data collection process and generated themes captured a broad range of perspectives and experiences.^27^

### 2.4 Analysis

We used line‐by‐line coding (organizing data into concepts and key phrases),^20^, ^22^ then focused coding and categorizing (coalescing large amounts of data into categories),^22^ and then theoretical coding (interpreting relationships between constructed categories and themes to provide insight into potential theories).^20^ We employed a constant comparative method within and across transcripts to elevate the analysis to a conceptual level.^20^ Data analysis was using the NVivo software.^28^

## 3 RESULTS

### 3.1 Participants

A total of 28 patients with CRC across Canada participated in the study with characteristics shown in Table 1. There were 10 participants (36%) in treatment, 12 participants (43%) in follow‐up, and 6 participants (21%) in the beyond phase.

**TABLE 1:** Participant characteristics.

	*N* (%)
*Demographics characteristics*	
Sex	
Female	19 (68)
Male	9 (32)
Gender identity	
Woman	19 (68)
Man	9 (32)
Sexual orientation	
Homosexual	1 (4)
Asexual	1 (4)
Bisexual	2 (7)
Heterosexual	24 (85)
Race	
White	19 (68)
Black, Afro‐Caribbean or African American	3 (11)
East Asian	2 (7)
Central Asian	1 (4)
South Asian	1 (4)
West Asian	1 (4)
Indigenous (e.g., First Nations, Metis or Inuk (Inuit))	1 (4)
Ethnicity	
Canadian	18 (64)
Canadian, American	2 (7)
Canadian, French	2 (7)
Irish	1 (4)
Scottish	1 (4)
English, Korean	1 (4)
Hawaiian	1 (4)
Oneida, Shishalh (Sechelt), Tseshaht	1 (4)
Saulteaux	1 (4)
Canadian province/territory of residence	
British Columbia	17 (60)
Ontario	8 (29)
Alberta	2 (7)
Yukon	1 (4)
*CRC Characteristics*	
Age at diagnosis (range)	
18–49	19 (68)
50–79	9 (32)
Cancer stage at diagnosis	
Stage I	1 (4)
Stage II	11 (39)
Stage III	8 (28)
Stage IV	8 (28)
Cancer site at diagnosis	
Colon	15 (54)
Rectum	5 (18)
Both	8 (28)
Phase of care at time of interview	
Treatment	10 (36)
Follow‐up	12 (43)
Beyond	6 (21)

### 3.2 Theory

**FIGURE 1:**
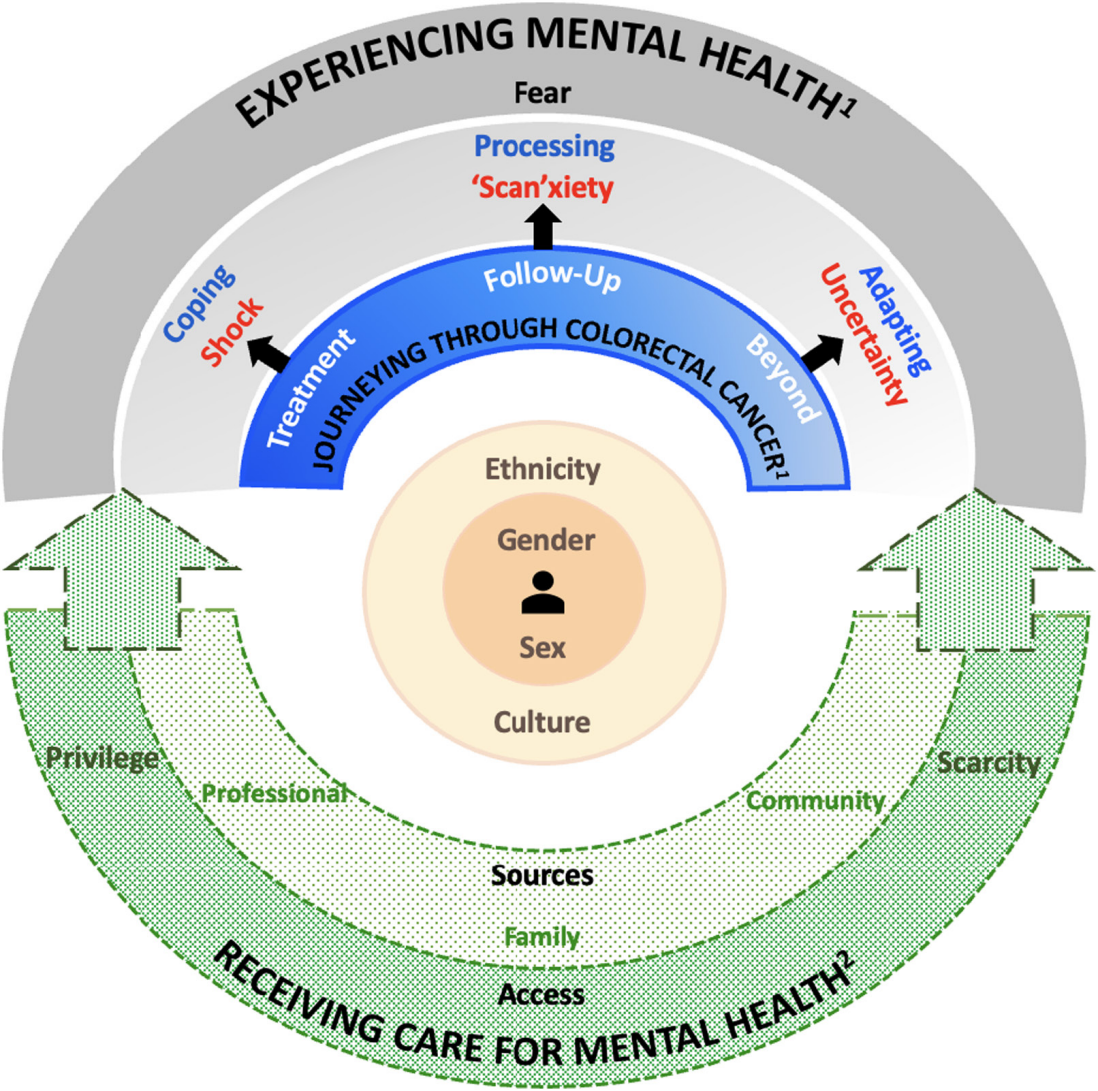
Mental health in people living with and beyond colorectal cancer: A patient‐oriented constructivist grounded theory. ^1^Solid colors and lines depict themes that are actualized and/or experienced by all participants, albeit in diverse ways. ^2^Dashed green arcs with dotted shadings represent mental health care, acknowledging that it may not be actualized or received by all participants.

Our qualitative analysis led to a patient‐oriented constructivist grounded theory exploring mental health experiences in patients with CRC throughout the care continuum (Figure 1). At the core of the theory is the theme of one's self‐identity, which captures contextual factors, such as gender, sex, ethnicity, and culture. Above self‐identity, the theme of journeying through CRC, spans phases of the CRC care continuum from treatment to follow‐up to beyond. Experiencing mental health is the theme that represents patients' emotional and cognitive‐behavioral responses, which are expressed differently, depending on where they are on the CRC journey. Underlying the complex interaction between self‐identity, journeying through CRC, and experiencing mental health is the theme of receiving care for mental health, which represents various supports as well as facilitators and barriers to access. Representative and supporting quotes for the themes describing mental health in the theory are presented in Table 2.

**TABLE 2:** Representative quotations for themes describing mental health in the theory.

Themes	Subthemes		Respective quotations
Experiencing mental health	Fear		“Going through the treatments, I always think about whether or not they're working […] I think about that all the time… the uncertainty and fear, that's what keep me up at night. I feel like the fear is never going to go away, it's just always on my mind.” (Participant 21, treatment)
			“Now that I'm past the treatment part, I'm better but there's always like the anxiety, like, you're scared the cancer is going to come back. I'm still being monitored, but with the statistics, I'm scared, I feel like it will come back. I just don't know when, if it's going to come back in five years, or in two years, or in ten years. Nobody knows.” (Participant 20, follow‐up)
	Emotional responses	Shock	“Getting diagnosed, it was a shock. I went into full on panic mode within myself.” (Participant 27, treatment)
			“I've always been in good health, so it was shocking… I spent two months pretty much lying in bed, just not really believing the diagnosis was real.” (Participant 18, treatment)
		‘Scan'xiety	“It's these tests… they really wreak havoc on the whole mental health. […] The constant worry and fear of what the results would be leading up to [the scans] really took a toll on my mental health and well‐being.” (Participant 19, beyond)
			The stress and worry are so high around those scans that it's very hard. Because it takes so long. It takes like three weeks to get confirmation. The fear of waiting, so you're just so anxious and miserable that you just want any closure as soon as possible. (Participant 25, follow‐up)
		Confusion	*“*The thing is they say you go back to life as normal. But that's not the case because to me, life changed after having gone through all this. And you don't know if it's going to happen again. When is it going to come back? It's always in the back of your mind. But everyone around me doesn't realize that. They all think I'm cured. Everybody thinks you're cured. That's it. They think you got to go back to live life as normal and how it used to be before. But things are not normal.” (Participant 11, beyond)
	Cognitive‐behavioral responses	Coping	“You don't necessarily have time to process what's happening to you when you're in active treatment.” (Participant 17, follow‐up)
			“We don't know how to process through this time [of active treatment] because we're just focused on trying to survive.” (Participant 26, follow‐up)
		Processing	“For many of us, this [follow‐up] phase is when we finally have the mental space to start thinking about ‘what happened to me?’ This is the time where we actually process our experiences. A time where I can actually recognize my emotions and feel the emotions that I was likely just trying to avoid before because I had no mental capacity or energy to address them before.” (Participant 24, follow‐up)
			“In this year following my treatment completion there's been a lot of processing and grieving for everything that did happen that I couldn't process while it was happening. I realized the roller‐coaster of a journey that I went through. […] I felt waves of anxiety and mood changes… all these changes to my mental health emerged when I was processing.” (Participant 25, follow‐up)
		Adapting	“There are some significant, permanent changes to my body system. There's definitely changes in what we used to consider normal for us. Even catching an early ferry, I'm anxious and don't really think I can do it because of frequency and urgency early in my days, which is new for me because of this cancer.” (Participant 15, beyond)
Receiving care for mental health	Sources	Processional	“The free ones from [the cancer care center] gives you six sessions and then they wash their hands after. But unfortunately, going through diagnosis like this is not a six sessions turnaround situation. So sessions were not that helpful, they were not enough. They were rushed which made it hard… they don't really get you to understand what your feelings really means. […] This needs to change within the system.” (Participant 13, follow‐up)
			“My GP was very compassionate. When I got the CRC diagnosis, she actually called me to see how I was doing. When I was hospitalized, she even came to visit me when she was on her shift. That was very nice and made a difference.” (Participant 21, treatment)
		Family	“It was just my family and immediate family have been very supportive… things were far too overwhelming, but my husband has been a rock. My mother, and my husband's parents as well… they have been a huge support, looking after a 5 year old and picking up slack, and that kind of thing. So I would say more so just family support kept me fairly mentally stable.” (Participant 12, follow‐up)
			“On the personal side, I have an incredible support system. So husband, daughter, sisters, friends. I could've made it through without them, but it would have been a lot harder. So definitely you can say my mental health has improved because I had these people along. I felt very supported by friends and family.” (Participant 10, follow‐up)
		Community	“Just knowing others and being in the same room with them is really therapeutic. Just knowing that everyone understands the particular kind of situation is really valuable and very validating… these groups even kind of normalized how I was feeling.” (Participant 25, follow‐up)
			“There's a lot of information, a lot of stories of support but also all the stories of everybody else's tragedies. So you need to be careful about going into those spaces for your own mental health, because it can be very depressing. It can be very hard.” (Participant 4, beyond)
	Access	Privilege	“The way our health insurance system is set up, it's a huge blocker for a lot of mental health services that gets partitioned into being covered. This is a piece that is very difficult for folks. Then you might just turn to other avenues, like private ones. But then there's the cost aspect that prevents a lot of the access. If you don't have access to these services, you won't even be opened to trying these services because you're just worrying that you can't possibly pay for them. This just contributes to the worry and the anxiety we already have, and sometimes the symptoms just get worse. So I definitely think the financial impact is a barrier to care.” (Participant 24, beyond)
			“So far, I've had great care. I have no negative comments about the care I've received. I've had a supportive [medical] team and my oncologist has provided me with the support I needed, even referring me to a social worker. I wouldn't have known there were [mental health] services available for me here if I wasn't referred. So he responded to my physical concerns and was also opened to listening to my mental health concerns. But I know a lot of other patients don't have the same experience as me. And I find hearing a lot of their cancer experiences very different than what I had because my experience has been very positive compared to theirs were quite negative.” (Participant 9, follow‐up)
		Scarcity	“Now that I'm done treatment, I have a lot of questions. But you can't get a hold of [healthcare providers] anymore. I've called the cancer care center and left many messages. The secretaries call back and will give you one‐ or two‐line answers. Nobody gives you answers. You just get left in the dark after. There was no follow‐up, but there has to be some sort of continued care, not just scans and tests every few months after you finish your treatment. There should be continued care from them about like, “this is where you can find resources to help with this and this symptom.” Or anything. They all just keep you so much in the dark about everything that it's even harder on your mental health once you start realizing how much they're keeping you in the dark.” (Participant 13, follow‐up)
			“The aftercare piece is really where you feel the healthcare system fall apart. Those mental health issues still exist for people but there's just not enough resources because you get cut off from the system and then you don't have access to things anymore. Because you can only see a psychiatrist when you're in active treatment, or you can only get a social work referral up until these many months since your last appointment. There are different kinds of system blockers. But this is the time when most people would start accessing mental health support because they have more time and energy to put into that realm.” (Participant 24, beyond)

#### 3.2.1 Theme 1: Journeying through CRC

Journeying through CRC captured the individual's experience with CRC and was comprised of phases of the CRC care continuum, which correspond to specific periods of treatment and/or care for CRC.

The treatment phase of CRC care commenced with a diagnosis, spanning until treatment completion. Patients in this phase were solely “focused on finishing” their CRC treatments, including surgery, chemotherapy, radiation therapy, immunotherapy, or a combination. They aimed to complete necessary treatments, achieving a point where cancer was inactive, varying based on individual circumstances, such as disease stage. After completing treatment, the follow‐up phase began, extending until no evidence of CRC or up to 5 years post‐treatment. In this phase, patients “prioritized continuously navigating these procedures,” including bloodwork, imaging tests, scans, and follow‐up appointments for surveillance aimed at detecting potential signs of cancer recurrence. Finally, the beyond phase began after a patient completes surveillance, pivoting their focus to “adjusting to significant, permanent changes to [their] body systems” and maintaining overall health. Notably, in contrast to the treatment and follow‐up phases, a patient's experiences during the beyond phase occur outside of the cancer care system.

#### 3.2.2 Theme 2: Experiencing mental health

The theme of experiencing mental health captured fear, which goes across all three phases of the CRC care continuum, as well as paired emotional (“feelings”) and cognitive‐behavioral (“activating thoughts” or “actions”) responses that correspond to each phase of care. A patient with CRC will experience these complementary responses consistently across all phases, with varying expressions. The paired emotional and cognitive‐behavioral responses comprised of the expressions: shock—coping (treatment), “scan”xiety–processing (follow‐up), and uncertainty—adapting (beyond).

##### Fear

Fear, a consistent response across all three phases of the CRC care continuum, emerges during the treatment phase when participants wrestle with the unpredictability of treatment outcomes, perceiving them as “out of [their] control.” Those currently undergoing treatment and reflecting on past experiences all expressed fear rooted in uncertainty. This fear revolved around apprehensions regarding treatment effectiveness and uncertainty about progress. Some feared “not knowing whether or not the cancer is gone” while others shared the “fear of not knowing whether the treatments are working enough to make the cancer go away,” significantly impacting their mental health.

During the post‐treatment phases (follow‐up and beyond), all participants experienced the second type of fear, where they were constantly “paralyzed by the fear of cancer recurrence.” Participants, spanning from 1 to 7 years post‐treatment, unanimously expressed persistent fears of detecting cancer recurrence. Despite the passage of time, participants in post‐treatment phases commonly experienced this fear, occupying their thoughts and emotions. Several in post‐treatment phases acknowledged the enduring fear of cancer recurrence, learning to “live with this fear of recurrence.”

##### Shock—Coping

Receiving a CRC diagnosis marked the beginning of the journey, and during the period between diagnosis and starting treatment, many experienced the emotional response of shock. Whether currently undergoing treatment or reflecting on their past experiences, participants expressed being “in shock and devastated after receiving the diagnosis.” Many described feeling completely taken aback, unable to comprehend the gravity of the situation. The feeling of shock profoundly impacted their mental health, leading to additional symptoms such as panic, worry, and distress. For some, the subsequent feelings of panic and worry were often due to grappling with the sudden realization of “what does the future look like now?” Others shared that the emotional impact of shock led to “a state of disbelief,” which was characterized by an initial refusal to accept their diagnosis. Depending on when treatments began, this period ranged from weeks to months, with many “refus[ing] to believe that the diagnosis was true.” The magnitude was heightened for those who had “always been healthy,” as the contrast between their prior perception of good health and the reality of a serious illness intensified their shock and disbelief.

During treatment, coping, the initial cognitive‐behavioral response, played a crucial role as participants engaged in diverse coping strategies to navigate their treatments. Many adopted a cognitive‐based coping strategy “focused on being in survival mode.” This involved directing their attention solely towards surviving and overcoming their CRC diagnosis, with participants emphasizing a sudden shift in behaviors, all “trying hard to survive” once treatments began. Several coped by “putting on a good mask to focus and get through treatment.” Others shared that amidst the demanding treatment process, “mentally focusing on survival in a way helped manage the initial shock of the diagnosis,” allowing them to compartmentalize emotions and channel energy exclusively towards enduring treatment. For some, this strategy involved engaging in essential treatment tasks, such as daily oral chemotherapy, scheduled radiotherapy, or sustaining a nutritious diet. This coping approach enabled participants to navigate treatment while preserving mental health, providing many with “some sort of purpose and direction.”

Despite the challenges of CRC treatments, all participants demonstrated unwavering determination to persevere, exhibiting this resolve through another cognitive‐based coping strategy of positive thinking. Actively focusing on generating positive and empowering thoughts, such as telling themselves to “try and be strong” or “focus on getting through each day” and that “[treatment] is just temporary,” helped participants regulate emotions and maintain a sense of control. For some, this strategy fostered greater confidence, serving as a source of motivation.

##### “Scan”xiety—Processing

After completing treatments, patients with CRC enter the follow‐up phase, marked by regular surveillance appointments involving physical exams and scans. Many participants expressed high levels of “worry and stress in the period before, during, and while waiting for their scan results,” terming this unwanted, overwhelming feeling as “scan”xiety. For participants, this anxiety‐inducing feeling led to panic and anxiousness during clinic visits. The unfamiliar medical settings, the association of scans with cancer recurrence, and the “fear of receiving potentially distressing news” all contributed to heightened “scan”xiety levels. Additionally, some expressed increased “scan”xiety levels due to the often lengthy for their scan results and reported this waiting period intensified their “scan”xiety levels, as they merely wanted to know “what's going on with [their] bod[ies] without delay.” While recognizing the importance of undergoing tests in detecting recurrence, participants also highlighted the negative impact of “scan”xiety on their mental health.

Processing, the second of the three cognitive‐behavioral responses, also coincided with the follow‐up phase, where participants began actively working through and “making sense of what happened to [them]” since their diagnosis. For most participants, the initial step in the processing phase involved reasoning, where they attempted to comprehend the implications of completing cancer treatment. Reasoning varied among individuals, with some sharing sentiments such as, “I couldn't comprehend it at first, what it meant the moment that I was done treatment.” They wrestled with the magnitude of what they had endured and sought to reconcile this with their newfound survivorship. Others used reasoning to “learn and understand the commitments and responsibilities that lay ahead,” including regular check‐ups and lifestyle adjustments. Engaging in this reasoning process enabled participants to comprehend their CRC experience and contemplate their future health and well‐being.

Particularly in the follow‐up phase, participants acknowledged suppressing emotions during treatment, experiencing an “emotional crash” and a surge of long‐suppressed feelings. This phase provided them the “mental space to reflect and process” their CRC journey. Engaging in this processing time provided an opportunity for self‐reflection, enabling them to revisit their CRC experience and confront the associated emotional and cognitive symptoms. For some, processing unveiled previously masked fear, anxiety, and depressive symptoms.

##### Uncertainty—Adapting

Experiences during the beyond phase were drawn from participants who had completed active treatment and surveillance for non‐metastatic CRC. These participants expressed uncertainties as they transitioned from frequent surveillance within the cancer care system to less‐frequent community/primary care. Although completed active treatment and surveillance, participants highlighted a contrast between the structured approach of treatment and surveillance and the lack of organization in the beyond phase. With lives “no longer revolving around scheduled appointments,” uncertainties about how to move forward became the prominent mental health expression in the beyond phase.

Adapting, the last of the three cognitive‐behavioral responses, also paralleled the beyond phase, where participants focused on maintaining their overall health and well‐being. Participants recognized that CRC significantly changed their lives, altering their understanding of “what is considered normal,” both physically and mentally. Physically, treatment effects prevented a return to the previous normal, necessitating an adjustment to a “new normal” way of functioning. For some, it was adjusting to changes in their bodies and energy levels, such as “constant fatigue”; for others, it was adjusting to new daily routines of work and recreation. Adapting took time, with participants noting “there [were] a lot of adjustments.”

Despite physical adaptations to the new normal, participants universally shared the experience of adapting to sudden changes in their mental health, ranging from abrupt feelings of fear and worry to occasional low moods. The emotional impact of CRC persisted into the beyond phase, leading to concerns about cancer recurrence or related health issues. To manage these emotions, some “learned to practice mindfulness” while others turned to “exercise to mentally alleviate concerns.” Gradually adapting physically and mentally, participants learned to embrace their new normal and coexist with the effects of their diagnosis.

#### 3.2.3 Theme 3: Receiving care for mental health

Participants' perspectives on receiving care for mental health was captured in another theme that described various sources of mental health care, as well as facilitators and barriers to access across the CRC care continuum. Unfortunately, mental health care was not received by all.

##### Sources

All participants commented on sources of mental health care and support, namely professional, family, and community. Participants shared how these sources uniquely impacted their overall mental health in both beneficial and adverse ways.

##### Professional

Participants viewed professionals as a source of mental health support, primarily during the treatment and follow‐up phases, with diverse experiences across public and private services.

A few participants were aware of professional mental health supports (i.e., counselors, social workers) from cancer centers or doctors within the Canadian public healthcare system during treatment. The majority were “unaware that [mental health] services [were] available to [them]” considering this a “huge gap in the system.” Some who utilized cancer center counseling services during treatment shared positive experiences. They highlighted the positive mental impact of “talking to someone separate from family and friends.” In contrast, several expressed dissatisfactions with cancer center services, citing frustration at the “limit of only five or six free sessions” with a social worker during treatment. They felt constrained to address all their mental health concerns within a handful of sessions.

Some participants received personalized mental health support from their general practitioners (GP), including frequent check‐ins, referrals to therapists, and tailored advice on prescribed antidepressant medications. One participant facing insomnia and anxiety during CRC treatment highlighted how “[her] GP would check in on her to make sure the as needed anti‐anxiety medications were working,” Another participant valued her GP's compassionate care, with hospital visits and phone calls during her CRC journey, providing emotional support.

A couple of participants in the post‐treatment phases opted for private mental health services, reporting positive experiences. They found private therapy sessions beneficial, “learning mindfulness techniques from [their] therapists” and received ongoing support. Those who used both cancer center and private mental health services shared that, “the most helpful, unfortunately, is anything that you pay for.”

##### Family

Participants described “constant support from family” throughout the CRC care continuum, which uplifted their mental health. The support was in the form of receiving financial assistance, accompanying to medical appointments, and assisting with daily activities. Family members, constantly present, also frequently encouraged participants to persevere, engaging in open conversations and actively “listen[ed] to their concerns and reassure [their] worries.” These positive interactions left participants feeling optimistic and hopeful about navigating their CRC journey, providing them with emotional strength.

##### Community

Participants considered the community, particularly peer support groups, discovered independently or through patient referrals, as another source of mental health support across all phases.

Several participants emphasized the positive impact of peer support groups on their mental health. They valued learning from others' experiences, gaining insights about CRC and “learn about different treatment options available that others have gone through.” Describing these groups as supportive communities, participants found them therapeutic, providing a safe space to “share, seek advice, and even complain with other patients who were going through the same CRC journey,” both in‐person and virtually. Many felt empowered by peers in support groups, as witnessing others successfully navigate their CRC journey gave them the confidence that “I can do this too.”

While peer support groups positively impacted participants, many faced challenges leading them to “leave these groups.” Several viewed peer support groups as a “double‐edged sword”, providing information on “the good, the bad, and the ugly” aspects of CRC. Specific posts in online groups triggered fears for some, while others found it emotionally challenging to “witness others go through years of recurrence” and negative post‐treatment effects. As a result, participants had mixed impressions of prolonged support from peer support groups, learning to navigate and recognize when these groups benefited or deteriorated their mental health.

##### Access

An important aspect of receiving mental health care is having access. This category is described by two factors—privilege and scarcity. These factors significantly influenced whether participants were able to access care for mental health through their CRC journey.

##### Privilege

Privilege refers to the unearned advantage or benefit that certain individuals have. Across all phases, participants' access to mental health care was impacted by various types of privilege related to economic status, education, and care, with each type acting as a facilitator or barrier. Unfortunately, access to mental health care is not equal across the population.

###### Economic privilege

Several participants lacked economic privilege to access mental health services, relying on the public healthcare system due to limited financial resources or private insurance. However, barriers persisted in the public system, including limited flexibility in choosing counselors and therapists, as well as long wait times causing detrimental impacts on mental health. Scheduling mental health care appointments at cancer centers also posed a common issue for participants accessing public healthcare, as several attempted to schedule these appointments multiple times without success.

In the follow‐up phase, several sought mental health services through the public system but found limited coverage under public health insurance. As a result, they explored alternatives, including private providers. However, this option proved to be financially unfeasible, as the “cost of [private mental health] services were simply beyond [their] means,” leaving many with no viable alternative but to rely on the limited range of services covered by the public system. This financial barrier compounded emotional symptoms, amplifying disparities in mental health care access.

Contrastingly, a few participants stated having private insurance, which facilitated their access to mental health care by circumventing the public system challenges. This advantage allowed them to choose providers who aligned with their needs and preferences, and schedule appointments effortlessly. This privilege positively impacted their mental health by enabling access to optimal care.

###### Education privilege

Participants with a healthcare or professional background had an advantage in accessing reliable and relevant mental health information. Participants with healthcare backgrounds efficiently accessed mental health services and identified supportive care providers. Their familiarity with the healthcare system facilitated self‐advocacy, effective communication with providers, and access to additional mental health support from their healthcare colleagues. This privilege made mental health care more accessible and manageable for these participants. Furthermore, those with a professional background, including as high school teachers, university professors, and government employees, highlighted how their “education level affected [their] ability to know how and where to go look for resources and support.” Despite limited mental health resources from providers or cancer centers, they independently used their research skills to seek online resources, self‐advocating for their physical and mental health. By critically evaluating such information, these participants felt reassured and validated, positively impacting their mental health.

Without strong healthcare system knowledge or professional background, several participants expressed frustration about the lack of public awareness about mental health services. They highlighted that their GP or oncologists had not offered referrals, and several emphasized they “did not know where or how to start looking for [information].” As a result, several felt overwhelmed by navigating the healthcare system and lacked the skills to advocate effectively for themselves.

###### Care privilege

Participants' experiences with mental health care were closely related to their perceptions of the quality of CRC care they received. Care privilege, representing disparities in CRC care, acted as a facilitator and a barrier to accessing mental health care. Some participants expressed feeling “well supported by [their] oncologist and medical team,” sharing they had oncologists and/or GPs who showed concerns about their mental health through regular check‐ins or referrals to specific mental health services. Rather than dismissing their concerns, these participants expressed they felt “lucky to have physicians who were empathetic and responsive to all my health needs.” These individuals emphasized the importance of having empathetic and responsive physicians, creating a safe environment to openly discuss mental health struggles with them.

However, several other participants found care privilege as a barrier to accessing mental health care, sharing that their medical teams showed limited concerns for their mental health during check‐ins, and did not provide participants with resources or referrals to mental health services. This led to missed opportunities for timely mental health care, as some participants were unaware that mental health could be a health challenge throughout their CRC journey. These participants reported that while physical health concerns were acknowledged, mental health concerns were often “shrugged off by [their] doctors” and created a hostile environment. This dismissal was disheartening, especially for some who “took a lot of effort to finally share [their] mental health struggles” and made them feel they “had to be strategic with who they shared their feelings with.”

The intersection of care privilege with race, age, and gender was particularly pronounced among certain participants. All self‐identified BIPOC (Black Peoples, Indigenous Peoples, and Peoples of Color) participants perceived a lack of consideration for their mental health concerns by healthcare providers, acting as a barrier to access. Having felt neglected and misunderstood at the hospital due to race‐related issues in the past, several BIPOC participants hesitated to discuss mental health concerns with providers, with all BIPOC participants expressing sentiments such as, “…I think because of my race, no one paid attention to me at the hospital.” In contrast, a few white participants shared how care privilege facilitated their access to overall health care, noting “the advantages being a white person moving through the system afforded [them] a certain level of privilege in care compared to others.”

Experiences of early‐age onset CRC (EAO‐CRC; diagnosed age < 50 years old) women participants also highlighted care privilege, with several facing dismissals of their physical and mental health concerns by healthcare providers based on age and gender. EAO‐CRC participants expressed frustration, with one sharing, “I felt completely left in the dark from the medical team since the beginning because I was told I was ‘a woman, who was too young and too healthy.’ […] And even now, I have so many questions after treatment….” Other EAO‐CRC women participants shared similar challenges, emphasizing how the intersection of gender and age created additional barriers to accessing quality healthcare. Facing dismissal and skepticism from providers, they all expressed anxiety and frustration over a year‐long struggle to receive referrals and appropriate care.

##### Scarcity

Across all three phases, participants' access to mental health care was also impacted by scarcity, specifically, the lack of: appointment time, continuity of care, and provider–patient education. In contrast to privilege, which may facilitate or impede access, the scarcity of these components all acted as a barrier to accessing mental health care.

###### Appointment time

In the treatment and follow‐up phases, participants mentioned experiencing “rushed appointments” with their healthcare providers, describing appointments as “very fast‐paced and business‐like.” Limited appointment time left participants feeling that healthcare providers focused solely on physical concerns, neglecting mental health concerns. Several reported that despite summoning the courage to raise their mental health concerns during appointments, they received similar responses from providers: “they're too busy. That's what [we] get back… they're too busy.” Consequently, many became increasingly hesitant to raise their mental health concerns, perpetuating the lack of attention to mental health throughout the CRC care continuum.

###### Continuity of care

Throughout post‐treatment phases, participants highlighted a scarcity of continuity of care. Despite the emergence of mental health challenges and physical side effects during follow‐up, participants felt a lack of support, care, and guidance from healthcare providers during this crucial period. Those who attempted to seek assistance revealed a disconnection from the cancer center post‐treatment, reflecting a lack in continuity of care that left participants disappointed with limited support for various physical and mental health concerns, including bowel movement discomfort and feelings of anxiety. Thus, multiple participants reported feeling detached from cancer centers during follow‐up, with some expressing “feeling abandoned in survivorship” and struggled to access needed mental health care. Challenges accessing mental health services and a lack of awareness about available resources were common, preventing participants from seeking support during this opportune time. Moreover, frustration arose as many realized mental health services available at cancer centers during treatment were no longer accessible to them post‐treatment. The limited resources for mental health support post‐treatment revealed a systemic gap in aftercare, despite persistent mental health challenges among participants.

###### Provider–patient education

Throughout treatment and follow‐up phases, participants reported a scarcity of education from providers on CRC and mental health, leaving many unaware that emotional and cognitive symptoms related to mental health were reasonable. While several acknowledged that the primary focus of their “providers [were] to treat [their] cancer,” and “address physical health,” they were surprised and disappointed at the lack of education about what to expect during their CRC journey overall. CRC participants in treatment stated that their healthcare providers did not explicitly inform them or acknowledge that their CRC journey could pose mental health challenges. Similarly, in the follow‐up phase, many revealed that they “had no idea what to expect after treatment, especially when it came to potentially experiencing mental health struggles.” Many expressed that lack of education was a barrier, hindering them from recognizing the magnitude of their mental health symptoms and understanding the importance of seeking help until much later. Despite experiencing symptoms such as “feelings of intense anxiety” and “mood dips for long periods of time,” many in the follow‐up phase did not seek help, attributing it to a belief that “[their] symptoms were not serious enough to ask for help.” Some shared that they “didn't even know where to start… or what supports [were] available and suitable for how [they were] feeling.” This lack of patient education acted as a significant barrier to accessing timely mental health care.

#### 3.2.4 Theme 4: Self‐identity

Certain contextual factors related to a participant's self‐identity, specifically gender/sex, ethnicity and culture, uniquely influenced their CRC journey and their mental health experiences.

*Gender/sex:* Gender expectations referred to participants' internalized beliefs about how they should behave and express themselves, which were influenced by sex, that is, biological functions.

Internalized gender expectations influenced participants' behaviors as men or women. Several men felt societal pressure to remain stoic and suppress emotions during difficult times as they have been “taught to act that way.” This belief impacted their behavior, with one participant acknowledging the pressure to “bottle up [his] emotions and just plow through, because [he's] supposed to resilient and steadfast.” This adherence to societal expectations of masculinity led some men to be resistant to discussing mental health struggles related to CRC, negatively impacting their mental health. In contrast, a couple of women participants demonstrated emotional expressiveness, a behavior associated with femininity. They mentioned that they were accustomed to expressing their emotions (e.g., “for women, I feel like we all tend to talk more because in a way we know that helps get our feelings out”). It appeared that women participants benefited from expressing their emotions and felt more comfortable to discuss and access support for their mental health.

Participants commented on how their sex, specifically biological functions, impacted their gender‐related behaviors, thereby influencing their emotional responses. Several male participants expressed the “fear of losing normal body functions,” which they perceived as essential to their masculine behaviors, including “being confident” and “strong.” Consequently, changes or decline in physiological functioning (e.g., urinary incontinence) due to treatments caused anxiety, stress, and insecurity in several males. They perceived physiological functioning as crucial for their “overall confidence,” and the fear of losing these functions significantly impacted their confidence, work, and recreation. Relatedly, biological functions, specifically menstruation and reproductive abilities, positively influenced females to express their emotions as women. Female participants noted that “managing emotional changes with hormonal fluctuations every month” normalized emotional variability, making them more comfortable expressing emotional responses related to CRC. This normalization positively impacted some women, fostering a willingness to “openly talk about [their] feelings.” Additionally, having experienced reproductive events, females were more accustomed to discussing difficult aspects of their bodies, positively impacting their mental health amidst physical challenges.

*Ethnicity and culture*: An interdependent relationship emerged between, ethnicity, which represented participants' self‐identification based on culture, that is, shared ideas, beliefs, and values.

Participants, representing diverse ethnicities, such as Canadian, BIPOC and European as well as various cultural backgrounds, including Indigenous, Asian, and European cultures, shared experiences influenced by cultural norms that practiced emotional restraint. Across the cultural identities, mental health was a stigma (e.g., “we were taught to never express an emotion or share that we're struggling”) which promoted on silence around mental health and posed challenges for individuals in discussing their mental health with others. Moreover, BIPOC participants from Indigenous and Asian cultures shared the belief that mental health is a personal responsibility. This cultural perspective contributed to self‐blame, exacerbated mental health struggles and impacted participants' approach to mental health care, with some feeling obligated to manage and “deal with their anxiety struggles [on their own].” Several BIPOC participants avoided seeking support for their mental health struggles, viewing it as a sign of weakness. This avoidance was further compounded by the perception that mental health supports, such as “therapy, is not encouraged or respected,” in certain Indigenous and Asian cultures.

## 4 PARTICIPANT CONSIDERATIONS

In addition to the theory on mental health, findings also encompassed participants' considerations, where they shared their desires on the “could haves/should haves.” Given the challenges to mental health care access, participants emphasized the need to reassess mental health care approaches and reevaluate resource distribution, advocating for timely and impactful information.

Participants recommended initiating mental health care with “verbal check‐ins,” (e.g., “it would've been nice just to be asked a simple ‘how have you been feeling?’”) from providers, to acknowledge the mental health aspect of the CRC journey. Early recognition of mental health in the CRC journey is crucial, as this could lead to conversations about available supports, normalize experiences, and prevent feelings of isolation. Moreover, participants reported varied experiences in the timing and content of mental health resources, with some sharing “receiv[ing] a few pamphlets on the day of diagnosis” and others recalling that they “weren't provided with any pamphlets or resources from anyone.” To address this, an exploration of the types (online or physical copies) and timing of resource distribution is necessary. Participants emphasized the importance of providing impactful and useful information in the right format and at the right time, with suggestions for a “follow‐up care package” after treatment from cancer centers containing mental health support information for patients to consult and access. This suggests that patients may be more ready for such resources during the follow‐up phase when they are processing their emotions and mental health.

## 5 DISCUSSION

Our study is the first to explore the lived mental health experiences of patients across the CRC care continuum. Using a patient‐oriented constructivist grounded theory, we explored mental health experiences of patients with CRC across treatment, follow‐up, and beyond. Mental health experiences encompass various emotional and cognitive‐behavioral responses which were expressed differently, depending on the phase of care. Our theory also illustrated that underlying mental health experiences, were individuals' experiences with mental health care. Mental health care experiences involved a range of supports, including factors that either facilitated or impeded access to care. Despite a broad spectrum of care experiences, many individuals faced unmet mental health care needs. Notably, contextual factors of one's self‐identity uniquely influenced their mental health experiences. Overall, our findings have implications to support a patient‐centred approach to addressing mental health for patients with CRC.

Despite evidence of increased mental health risks in patients with CRC^1^, ^2^, ^29^, ^30^ and its impact during cancer treatment and recovery,^31^, ^32^ research on mental health experiences throughout the CRC care continuum remains limited. However, aspects of our findings are consistent with previous qualitative studies that have explored patients with CRC and their general experiences throughout specific stages of cancer care.^9^, ^10^, ^11^, ^12^, ^13^, ^14^ Although these studies investigated overall patient experiences, some have reported themes alluding to aspects of mental health. A 2018 grounded theory study found that all 24 participants experienced distress preoperatively and operatively,^11^ while another 2019 grounded theory study highlighted the use of coping mechanisms to manage emotional distress, including depression, anxiety, and fear during surgical treatment.^10^ Similarly, we also found that during the treatment phase, patients with CRC used various cognitive‐based coping strategies to navigate their treatments, with coping being the primary cognitive‐behavioral response in this phase. Additionally, a 2009 phenomenological study found that in the postoperative stage, patients' emotional state initially mirrored their physical condition until their awareness of having cancer resurfaced, leading to fear and anxiety.^12^ This parallels our findings in the follow‐up phase, where participants, after completing treatment, finally had the time and space for emotional processing and self‐reflection. For some, processing led to the uncovering of previously masked fear, anxiety, and depressive symptoms. Lastly, mental health support, particularly social support, has been identified to improve health outcomes for patients with CRC.^33^ A 2012 qualitative study revealed that social support from family and friends greatly enhanced patient well‐being,^34^ which align with our findings, as participants also viewed family as the most constant source of mental health support that contributed positively to their mental health.

Our study revealed that patients' mental health experiences involve emotional and cognitive‐behavioral responses, shaping their interactions with mental health care. Understanding these responses prompt us to advocate for health care providers to address mental health topics, including initiating conversations about potential symptoms across the CRC care continuum. As patients often overlook mental health resources during treatment, providers can play a crucial role by addressing mental health topics during follow‐up. Previous studies among breast and CRC patients in survivorship have shown that discussing both physical and mental symptoms and concerns with health care providers in the post‐treatment phases offers reassurance about cancer recurrence and aids in the transition to life beyond cancer.^35^, ^36^ A 2022 cross‐sectional study found that 34% post‐treatment CRC participants emphasized the need for ongoing emotional support to manage concerns about cancer recurrence,^37^ aligning with our findings where fear of cancer recurrence was a consistent experience across all three phases of the CRC care continuum. Literature suggests that effective communication with health care providers about potential physical and mental health concerns is particularly relevant in CRC, due to the unique impacts of this disease.^38^ Furthermore, given the long‐term impacts and risks associated with untreated mental health concerns,^39^ our findings highlight the importance of providing ongoing patient support and education on mental health to empower patients with CRC to seek timely care throughout all phases of their CRC journey. Consistent with existing literature,^40^, ^41^, ^42^ our study demonstrates that patients who are well‐informed by their healthcare providers experience a greater sense of comfort and empowerment, enabling them to take responsibility for their well‐being and access appropriate mental health services. Therefore, integrating patient education on mental health and continuous support from healthcare teams throughout the CRC care continuum must be considered. For example, our participants recommended initiating mental health care conversations through short “verbal check‐ins” from providers to acknowledge the mental health aspect of the CRC journey. We further recommend that these educational efforts and conversations to be inclusive, considering diverse backgrounds and people of color with varied race, ethnic, and cultural experiences. Addressing these factors is crucial in encouraging individuals from diverse communities to seek necessary mental health support.

The strengths and limitations of our study warrants discussion. Collaborations with researchers, oncologists, and patient partners strengthened our data collection, analysis, and interpretation. While videoconferencing facilitated diverse perspectives from the Canadian CRC population, recruitment was limited to those with access to the required technology. Our data collection was enriched through using social media to capture and facilitate interviews representing a range of CRC and mental health experiences, spanning age, race, ethnicity, and cancer stage across all phases. However, we acknowledge the limitation of potential volunteer bias from such recruitment methods, as participants on social media platforms may possess unique characteristics. We also recognize that the overall sample size was influenced by participants' willingness and availability to participate in the study. Notably in our study, no recurrent CRC were observed, and participants with stage IV metastatic CRC did not experience unresolved or refractory CRC. Thus, based on the participants who participated as well as the phases that they were in at the time of their interviews, our theory may be interpreted from a curative care perspective. However, this reflects how our data and theory emerged, suggesting that future research on mental health could be explicit about whether they intend to use specific frameworks (e.g., curative‐intent, palliative‐intent) and aim to capture those specific patient experiences. While our study took a broad approach to understand mental health experiences in individuals with CRC, future research could explore these experiences within specific treatment contexts, such as recurrent cancer or palliative care.

## 6 CONCLUSION

Our study provided a comprehensive understanding of the mental health experiences of patients with CRC throughout the CRC care continuum. Understanding patients' emotional and cognitive‐behavioral responses and care experiences, can help identify opportunities for mental health supports and interventions for patients with CRC.

## AUTHOR CONTRIBUTIONS

**Vicki Cheng:** Conceptualization (equal); data curation (lead); formal analysis (lead); investigation (lead); methodology (equal); project administration (equal); validation (equal); visualization (equal); writing – original draft (lead); writing – review and editing (lead). **Helen McTaggart‐Cowan:** Conceptualization (supporting); formal analysis (supporting); supervision (supporting); validation (supporting); writing – original draft (supporting); writing – review and editing (supporting). **Jonathan M. Loree:** Conceptualization (supporting); supervision (supporting); validation (supporting); writing – review and editing (supporting). **Rachel A. Murphy:** Conceptualization (supporting); supervision (supporting); validation (supporting); writing – review and editing (supporting). **Mikaela Barnes:** Conceptualization (supporting); validation (supporting); writing – review and editing (supporting). **Haydn Bechthold:** Conceptualization (supporting); validation (supporting); writing – review and editing (supporting). **Norman Jansen:** Conceptualization (supporting); validation (supporting); writing – review and editing (supporting). **Mary A. De Vera:** Conceptualization (lead); formal analysis (supporting); funding acquisition (lead); methodology (lead); project administration (lead); supervision (lead); validation (equal); visualization (equal); writing – original draft (supporting); writing – review and editing (supporting).

## FUNDING INFORMATION

This research was supported by a Project Grant from the Canadian Institutes of Health Research, “Examining the epidemiology, treatment, and outcomes in young‐onset colorectal cancer” (Funding reference number: PJT‐159467). The funder had no role in study design, data collection and analysis, decision to publish, or preparation of the manuscript.

## CONFLICT OF INTEREST STATEMENT

The authors declare no conflict of interests.

## ETHICS STATEMENT

This study was approved by the Behavioral Research Ethics Board at the University of British Columbia (H22‐01319).

## PATIENT CONSENT STATEMENT

Patient participation in the interviews were completely voluntary. Online consent forms were reviewed and obtained from all participants prior to participation. Oral consent was obtained again virtually prior to the interview to confirm the participants' consent.

## PRECIS

Our patient‐oriented theory reveals the dynamic interplay between self‐identity and mental health experiences across the CRC care continuum, encompassing diverse emotional and cognitive‐behavioral responses. Insights from this theory inform understanding of patients' mental health responses and experiences and guide the integration of mental health into CRC care.

## Data Availability

The data underlying this article will be shared on reasonable request to the corresponding author.
